# The Laccase Gene Family Mediate Multi-Perspective Trade-Offs during Tea Plant (*Camellia sinensis*) Development and Defense Processes

**DOI:** 10.3390/ijms222212554

**Published:** 2021-11-21

**Authors:** Yongchen Yu, Yuxian Xing, Fengjing Liu, Xin Zhang, Xiwang Li, Jin Zhang, Xiaoling Sun

**Affiliations:** 1 Tea Research Institute, Chinese Academy of Agricultural Sciences, No. 9 South Meiling Road, Hangzhou 310008, China; yuyongchen@tricaas.com (Y.Y.); xingyuxian@tricaas.com (Y.X.); xinzhang@tricaas.com (X.Z.); lixiwang0392@tricaas.com (X.L.); 2 Key Laboratory of Tea Biology and Resources Utilization, Ministry of Agriculture and Rural Affairs, No. 9 South Meiling Road, Hangzhou 310008, China; 3 Tea Research Institute of Fujian Province Academy of Agricultural Science, Fuzhou 355015, China; fengjingliu@163.com

**Keywords:** *Camellia sinensis*, laccase gene family, trade-off, development, induced defense, insect herbivore

## Abstract

Laccase (LAC) plays important roles in different plant development and defense processes. In this study, we identified laccase genes (*CsLAC*s) in *Camellia sinensis* cv ‘Longjing43′ cultivars, which were classified into six subclades. The expression patterns of *CsLAC*s displayed significant spatiotemporal variations across different tissues and developmental stages. Most members in subclades II, IV and subclade I exhibited contrasting expression patterns during leaf development, consistent with a trade-off model for preferential expression in the early and late developmental stages. The extensive transcriptional changes of *CsLAC*s under different phytohormone and herbivore treatment were observed and compared, with the expression of most genes in subclades I, II and III being downregulated but genes in subclades IV, V and VI being upregulated, suggesting a growth and defense trade-off model between these subclades. Taken together, our research reveal that *CsLAC*s mediate multi-perspective trade-offs during tea plant development and defense processes and are involved in herbivore resistance in tea plants. More in-depth research of *CsLAC*s upstream regulation and downstream targets mediating herbivore defense should be conducted in the future.

## 1. Introduction

As sessile organisms, plants cannot escape adverse biotic and abiotic stresses that have negative impacts on their growth and development. Under these pressures, plants have evolved a sophisticated network of defenses over millions of years [1]. In general, plant defenses can be classified as constitutive, which are always present, and induced, which are activated only upon attack [2]. In terms of induced defense, plants perceive and decode damage-associated and/or herbivore-associated molecular patterns (DAMP/HAMP) via receptors and then activate early signaling components such as Ca^2+^ influx, reactive oxygen species, and MAP kinases. Subsequently, specific defense responses are initiated and regulated by multiple phytohormone signaling pathways, including jasmonic acid (JA), salicylic acid (SA), ethylene (ET), hydrogen peroxide (H_2_O_2_) and other growth-related phytohormones, such as gibberellins, cytokinins, brassinosteroids and auxins [3]. Cross-talk between these signaling pathways activates a series of changes at the molecular, biochemical, and physiological levels, which may lead to the enhancement of plant resistance but the reduction of plant growth, development, and productivity [4]. Activation of defense responses generally comes at the expense of plant growth penalties, which could be in line with a delicate balance between growth and defense, known as the “growth-defense trade-off” phenomenon. Competition for limited available resources has long been considered as the force driving this trade-off, but it has recently been hypothesized to be the result of opposite molecular pathways regulating growth and defense [5].

Laccase (LAC) is a kind of polyphenol oxidase (PPO, EC 1.10.3.2) that occurs widely in bacteria, fungi, insects, and higher plants. Common LAC acting as multicopper oxidases, has four copper atoms, all forming their catalytic site, and contains three cupredoxin domains (domain 1, 2 and 3). One mononuclear copper site exists in domain 3 and one trinuclear copper cluster appears at the interface between domain 1 and 3. The domain 2 joins and positions domain 1 and 3 [6,7]. The important biological functions of LAC include the broad spectrum of substrate and using molecular oxygen as the final electron acceptor. Many studies have demonstrated that plant LAC play important roles, involving in polymerization of phenolic compounds, lignification of the cell wall structure, defense against various stresses, wound healing and iron metabolism, amongst others [8]. LAC catalyzed the final step of monolignol polymerization and involved in lignin biosynthesis, which has been demonstrated in LAC mutants. In *Arabidopsis thaliana*, *lac4* and *lac17* double mutants showed hypolignified fibres and collapsed xylem vessel phenotypes, and *lac4*, *lac17*, *lac11* triple mutants displayed severe growth defects and failed to exhibit lignification either in stems or roots [9,10]. Overexpression of rice *OsLAC10*, pear *PbLAC1* and cotton (*Gossypium hirsutum*) *GhLAC15* in *Arabidopsis* and overexpression of *GhLAC1* in cotton enhanced lignin accumulation [11,12,13,14]. Overall, these studies have demonstrated that LAC proteins were essential for lignin formation in plants. The LAC genes exhibited diverse temporal and spatial expression patterns in xylem, lignifying and non-lignifying tissues, which suggested that LAC genes might play certain roles in the development of roots, flowers and seeds. For example, *AtLAC15* functions in root elongation and proanthocyanidin polymerization from its monomer epicatechin, while *AtLAC8* regulates flowering and one *OsLAC* is involved in yield gains [15,16,17].

Plant *LAC*s are induced upon exposure to both biotic and abiotic stresses and are implicated in plant defensive processes. Overexpression of *GhLac1* in cotton enhanced broad-spectrum biotic defense against the fungal pathogen *Verticillium dahlia* and the insect pests cotton bollworm (*Helicoverpa armigera*) and cotton aphid (*Aphis gossypii*) via increased lignin deposition. Furthermore, suppression of *GhLAC1* leads to a redirection of defense metabolic flux in the phenylpropanoid pathway and JA accumulation to confer resistance to *V. dahliae* and cotton bollworm but potentiates susceptibility to cotton aphids [12]. The VIGS-based transient silencing of wheat *TaLAC4* increased susceptibility to *Fusarium graminearum*, mainly due to decrease pathogen-induced lignification of secondary cell walls [18]. Additionally, *ZmLAC1* can be induced by salt stress, suggesting its possible role in *Zea mays* tolerance to salt stress [19]. However, a high degree of redundancy of LAC genes has been reported in many plant species [20]; thus, investigating the gene family and screening function-specific genes would be helpful for genetic engineering, which is especially important for plants with a larger number of LAC genes.

The tea plant (*Camellia sinensis* (L.) O. Kuntze) originated in Southwest China and is one of the most important woody cash crops. The tender buds and leaves of tea plants are the raw material for commercial tea, the most widely drunk nonalcoholic beverage in the world [21]. In nature, tea plants suffer from abiotic stresses such as drought, extreme temperature, and biotic stresses such as herbivore infestation and pathogen infection, all of which incur severe losses of yield and low-quality tea production. Thus, there has been considerable attention focused on investigating how tea plants resist multiple challenges. Due to the multiple functions of plant *LAC*s as stated above and the few studies on tea laccase genes (*CsLAC*s), investigation of the *CsLAC* gene family is extremely necessary. In this work, based on a transcriptome and genome database of tea plants, 43 candidates of *CsLAC* family were identified and validated in *C. sinensis* cv ‘Longjing43′ cultivars using reverse transcription polymerase chain reaction (RT-PCR) and RACE-PCR methods. The identified *CsLAC*s were then further analyzed for gene architecture, conserved domain profile, and physical properties (protein size, isoelectric point and subcellular localization). Next, the expression levels of the total 43 *CsLAC*s in mature leaves were analyzed from RNA-seq datasets. Furthermore, using quantitative real-time PCR (qRT-PCR), the expression profiling of important *CsLAC*s in leaves at different developmental stages and different tissues were detected, and the transcriptional changes of *CsLAC*s under abiotic and biotic stresses were also examined. The present study helps us to better understand the multiple layers of biological functions of *CsLAC*s and provides theoretical references for studying the molecular mechanisms of LAC-mediated multiperspective trade-offs in tea plant during development and defense processes.

## 2. Results

### 2.1. Molecular Cloning, Phylogenetic Analysis and Chromosomal Distribution of CsLACs

**Figure 1:**
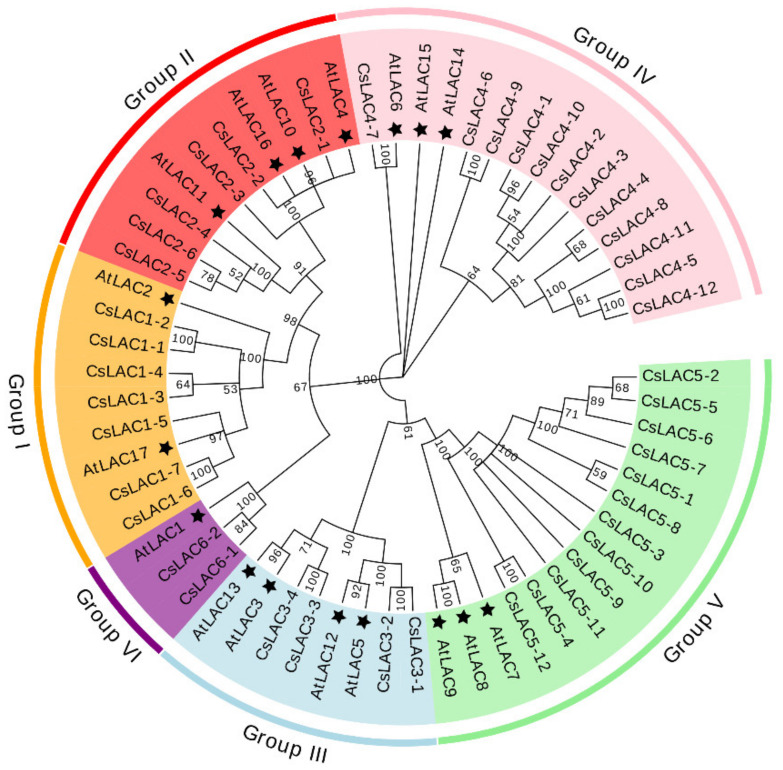
Phylogenetic analysis of *Arabidopsis* and *Camellia sinensis* LAC genes. Phylogenetic tree was constructed using 60 protein sequences from *Arabidopsis* (17) and *C. sinensis* (43). Neighbor-joining method was used with boot strap replication of 1000 times to create the phylogenetic tree. Six subclades of the family are highlighted in different colors. ★, *At*LAC1 to 17.

Through a genome-wide computational search (http://tpia.teaplant.org/Blast.html accessed on 30 November 2018) and subsequent gene model prediction, a total of 30 full-length *CsLAC*s were obtained using molecular cloning approaches, including 5′ and 3′ RACE and PCR amplification of coding regions, and the other 13 *CsLAC*s were identified from the second version of the tea genome (released in May 2020) [22]. A phylogenetic tree was constructed using the full-length amino acid residue sequences of 43 CsLACs and 17 AtLACs (Table S1). The 43 *CsLAC*s were divided into six subclades based on the classification standard of *AtLAC*s from *Arabidopsis* (Figure 1 and Figure S1). In detail, seven *CsLAC*s in subclade I were clustered in Group I with two *AtLAC*s, while six *CsLAC*s in subclade II were gathered in Group II with four *AtLAC*s. Interestingly, Group IV comprised 12 *CsLAC*s along with three *AtLAC*s. Subclade V contained 12 *CsLAC*s and was distributed together with stress-induced *AtLAC7*, *AtLAC8* and *AtLAC9*. In addition, Group III contained four *CsLAC*s and four *AtLAC*s with unknown functions. Group VI included two *CsLAC*s with only *AtLAC1*. These results showed that the LAC gene family in tea plants underwent specific evolutionary events after the divergence of *C. sinensis* and *Arabidopsis*.

**Figure 2:**
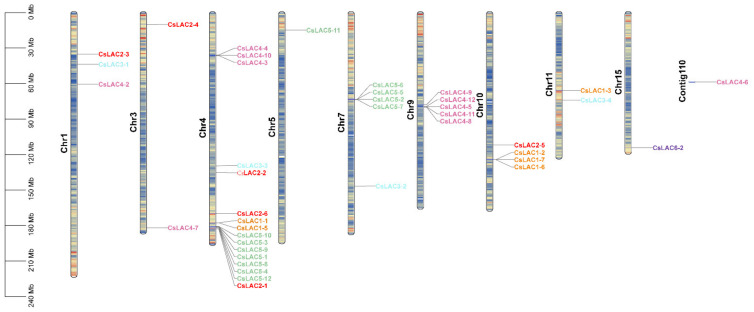
Chromosomal localization and clustering of *CsLAC*s in tea genome. Some chromosomes e.g., 4, 7 and 9 contain higher number of *CsLAC*s, but 5 and15 chromosomes each possesses only one *CsLAC*, presenting uneven distribution throughout the chromosomes.

The Tea Plant Information Archive provides the exact coordinates and orientations of *CsLAC*s in tea chromosomes. The identified 42 *CsLAC*s were randomly distributed on 10 out of 15 chromosomes, while the chromosomal locations of the remaining 1 *CsLAC*s remained unknown (Figure 2). In most cases, *CsLAC*s are present in distinct clusters on each chromosome. Chromosome 4 contained the highest number of *CsLAC*s (16), accounting for 36.3%, where seven *CsLAC*s from subclade V formed a cluster. A similar phenomenon was observed on chromosomes 7 and 9. Additionally, we found four *CsLAC*s on chromosome 10, 3 *CsLAC*s on chromosome 1, and two *CsLAC*s on chromosomes 11 and 3. However, chromosomes 5, 15 and contig110 contained the lowest number of *CsLAC*s, with one on each.

### 2.2. Analysis of Conserved Domains, Gene Architecture and Promoter Elements of CsLACs

**Figure 3:**
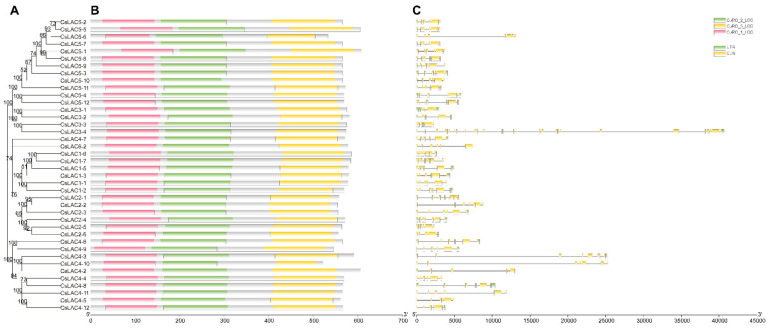
Phylogenetic tree of *CsLAC*s as well as protein conserved domain and gene structure of corresponding *CsLAC*s. (**A**) Phylogenetic tree of *CsLAC*s, as shown in Figure 1. (**B**) Conserved protein motifs and their distribution. The boxes with different colors represent the conserved motifs. (**C**) Exon-intron structure. The CDS, UTR (untranslated region) and introns are represented by yellow boxes, green boxes and gray lines, respectively.

We analysed the conserved domains of the CsLAC proteins and their distribution, as shown in Figure 3. All of the identified proteins (except for CsLAC4-1) shared three characteristic domains [NCBI CDD (conserved domain database): LAC, CuRO_1_LCC, CuRO_2_LCC and CuRO_3_LCC)]. The genomic and physical characteristics of 43 *CsLAC*s were further analysed in silico (Table 1). In total, the coding sequences ranged from 1425 to 1824 bp in length, as the exons of *CsLAC*s varied from 4 to 8, with 6.12 on average. The predicted molecular weights were 53.109 to 67.871 kDa, and the isoelectric points were 5.05–9.54. The theoretical pI values differed extensively among six subclades. All CsLACs in subclades I and II were basic proteins (pI, 8.2–9.5), while 10 of 12 CsLACs in subclade V and 7 of 12 in subclade IV were acidic proteins (pI, 6.0–6.8). In summary, these findings suggest the relatively conserved gene structure of *CsLAC*s and functional diversity either between different subclades (I, II and V, IV) or within the same subclade (IV).

**Figure 4:**
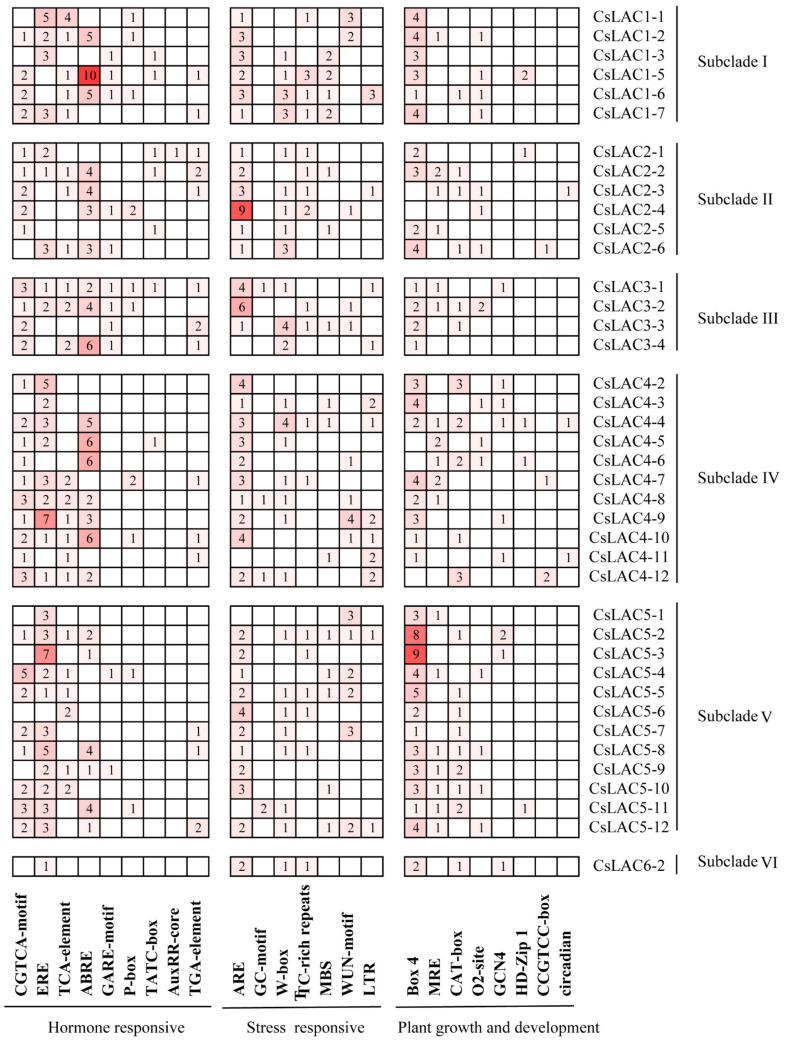
Analysis of *cis*-elements in *CsLAC* promoters. The numbers of *cis*-elements existed in the promoter sequences of *CsLAC*s were identified involving in three types biological processes. The depth of red colour indicates the quantity of *cis*-elements of *CsLAC*s.

*Cis*-regulatory elements are DNA sequences located in the promoter region of target genes and interact with transcription factors to trigger target gene expression. To gain insight into the regulatory functions of the *CsLAC* promoters, a number of *cis*-acting elements were predicted in the promoters of 40 *CsLAC*s. Many *ci*s-elements including the plant hormone responsive elements, stresse responsive elements and plant growth and development responsive elements were observed in the *CsLAC* promoters, and which were divided into three categories and 24 types (Figure 4). Among them, the hormone responsive types were especially abundant. For instance, the most abundant *cis*-elements were JA-responsive CGTCA-motif in the 31 *CsLAC* promoters, followed by ethylene-responsive EREs (30 *CsLAC*s), SA-responsive TCA-element (24 *CsLAC*s), abscisic acid-responsive ABREs (23 *CsLAC*s) and gibberellin-responsive elements, including GARE-motif, P-box and TATC-box. These results imply that *CsLAC*s may play significant roles in plant response to different phytohormones. The stress-related *cis*-elements were also found in the promoter region of *CsLAC*s. The most abundant *cis*-elements were anaerobic-inductive elements (ARE, *cis*-acting regulatory element essential for the anaerobic induction) in the 36 *CsLAC* promoters. In addition, W-box, TC-rich repeats (*cis*-acting element involved in defense and stress responsiveness), MBS (drought-inducible elements), WUN-motif (wound-inducible elements), LTR (low temperature-inducible elements) were identified in some *CsLAC*s. These results suggest that *CsLAC*s may be more generally involved in the responsiveness of different stresses. Moreover, the promoter regions of many *CsLAC*s contained different binding sites involved in plant development and stress responses. Out of 40 *CsLAC*s, 35 possessed Box 4 element (part of a conserved DNA module involved in light responsiveness), 18 possessed MRE element (MYB binding site involved in light responsiveness), and 20 had a CAT-box (*cis*-acting regulatory element related to meristem expression), which might be involved in plant development and stress responses.

### 2.3. Expression Patterns of CsLACs during Leaf Development and in Different Tissues

**Figure 5:**
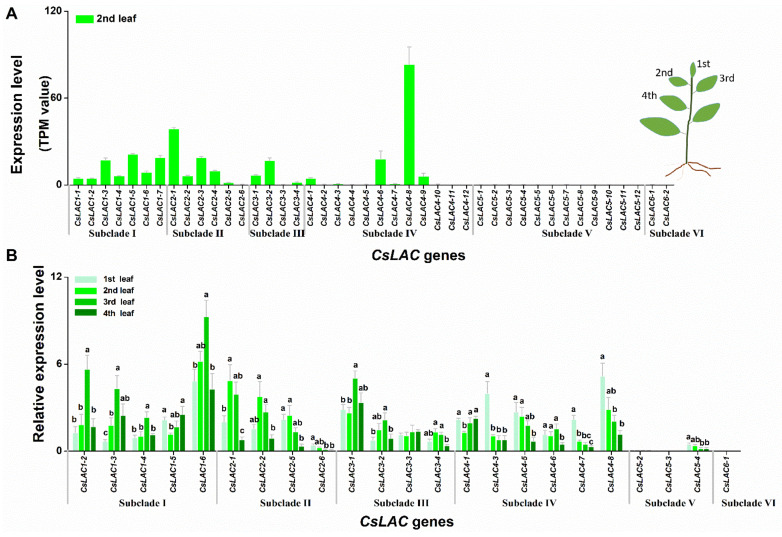
Expression patterns of *CsLAC*s in leaves of *C. sinensis* cv ‘Longjing43′ cultivars. (**A**) Expression levels of 43 *CsLAC*s in the 2nd leaves and their abundance was evaluated by converting read count to TPM. Inset: Schematic illustration of sample collection. (**B**) Expression patterns of *CsLAC*s in leaves at different stages of maturity (1st, 2nd, 3rd and 4th leaf). Different letters indicate significant differences among different maturity stages (*p* < 0.05, Turkey’s honest significant difference (HSD) post-hoc test).

Tea leaves are important raw material of tea products, containing numerous bioactive components that are responsible for the distinctive taste and fragrance [23]. Firstly, we analysed the expression levels of 43 *CsLAC*s in the 2nd leaf from the transcriptome database (Figure 5A). The results showed that the genes in subclades IV (except for *CsLAC4-1*, *CsLAC4-6*, *CsLAC4-8*, *CsLAC4-9*), V and VI were barely expressed, while genes in subclades I, II and III were highly or moderately expressed, exhibiting apparent differential expression patterns between these subclades. Then, by RT-qPCR, we investigated their development-specific expression patterns of *CsLAC*s during leaf development process (1st, 2nd, 3rd and 4th leaves, shown as a schematic in Figure 5A). Due to primer design restrictions and lower expression levels, only 23 *CsLAC*s were assessed in this study. Among these selected genes, the transcripts of nearly all members of subclade I (except for *CsLAC1-5*) gradually increased from the 1st to 3rd leaves and subsequently declined in the 4th leaves. In contrast, the transcripts of all genes in subclade II gradually decreased from 2nd to 4th leaves. Subclade IV members, except for *CsLAC4-1* and *CsLAC4-6*, were highly expressed in the 1st leaves but reduced in pace with maturity. The mRNA levels of subclade III members were comparatively irregular. The genes in subclade V and VI were at extremely low levels in leaves at all different stages of maturity, and these genes were therefore considered to be silencing genes, pseudogenes or inducible expression genes under stresses in the leaf tissue (Figure 5B).

**Figure 6:**
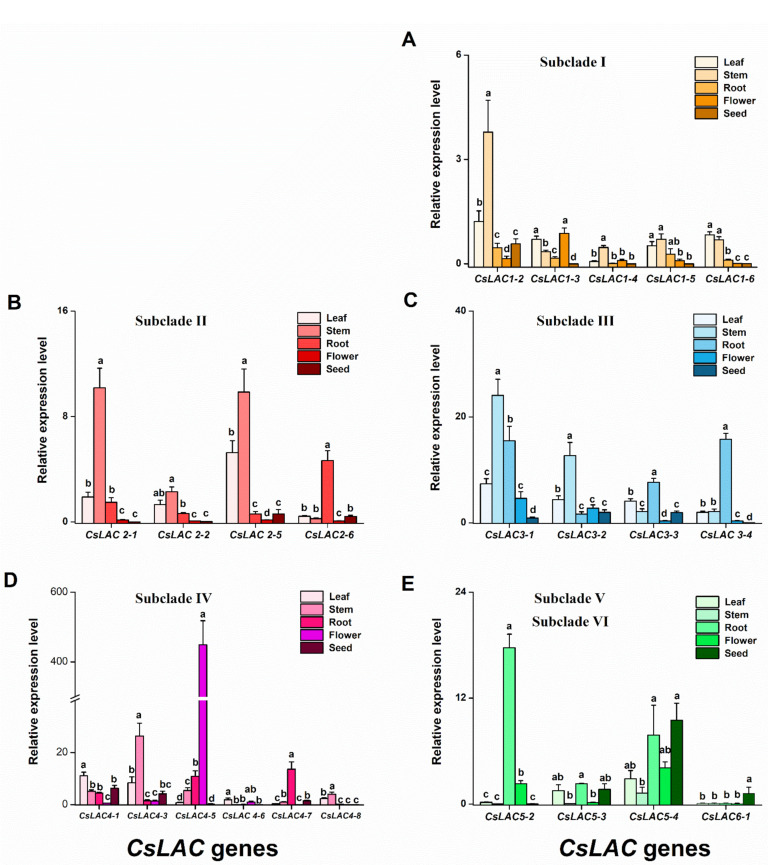
Transcript levels of *CsLAC*s in leave, stem, root, flower and seed of *C. sinensis* cv ‘Longjing43′ cultivars. The relative expression level of *CsLAC*s from subclades I (**A**), II (**B**), III (**C**), IV (**D**) and subclades V and VI (**E**). Results are expressed as mean +SE (*n* = 6). Different letters indicate significant differences among tissues (*p* < 0.05, Turkey’s honest significant difference (HSD) post-hoc test).

Lastly, the tissue-specific expression patterns of *CsLAC*s were detected by RT-qPCR. Generally, the expression of *CsLAC*s from subclades I, II, III and IV (except for *CsLAC4-5*, *CsLAC4-7*) showed the highest levels in leaves and stems, less in roots, and the lowest in flowers and seeds, whereas in subclades V and VI, *CsLAC*s (*CsLAC5-2*, *CsLAC5-3*) showed high or moderate expression levels in roots relative to other tissues. Some *CsLAC*s among the selected genes showed specific expression pattern. For example, only four *CsLAC*s (*CsLAC3-3*, *CsLAC4-1*, *CsLAC4-3*, *CsLAC4-7*) showed moderate expression in seeds. Two *CsLAC*s (*CsLAC5-4*, *CsLAC6-1*) were predominantly expressed in seeds, and *CsLAC4-5* was specifically expressed in flowers (Figure 6).

In general, *CsLAC*s within the same subclade showed similar expression patterns. Conversely, different subclades, e.g., subclades I, II versus subclades V, VI, showed differential expression patterns. These findings suggest that *CsLAC* familiy may play important and different roles in tea plant development, but the role of individual *CsLAC* may be limited.

### 2.4. Expression Profiles of CsLACs in Response to Exogenous Application of JA and SA

**Figure 7:**
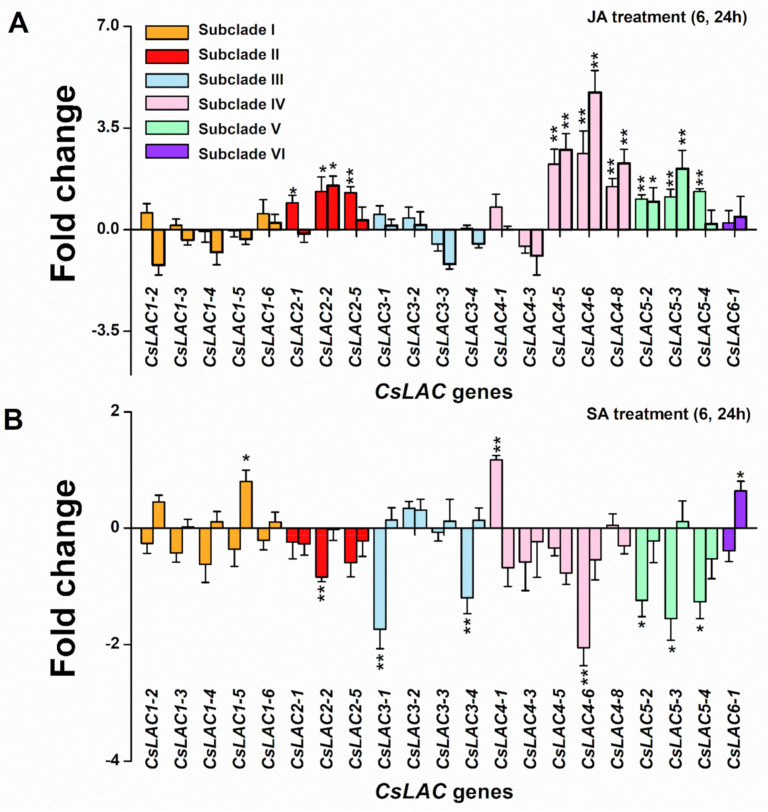
Expression patterns of differentially expressed *CsLAC*s from *C. sinensis* cv ‘Longjing43′ cultivars treated with jasmonic acid (JA) and salicylic acid (SA). The fold change in expression levels (treated group vs. control group) is shown on a log2 scale and the left and right bar graphs for each gene represent the fold change after 6h and 24h of treatment, respectively. (**A**) The change in expression levels of *CsLAC*s in response to JA treatment by RT-qPCR. (**B**) The change in expression levels of *CsLAC*s in response to SA treatment by RT-qPCR. The asterisks above lines indicate significant differences between the herbivore treatment and the control (*, *p* < 0.05, **, *p* < 0.01, Student’s *t*-test).

Plants respond to environmental stresses via the production of specific defense responses, which are regulated by multiple signaling pathways, including JA, SA, ethylene (ET), etc. Since JA and SA play important roles in the induced defense response of plants, including tea plants [24]. We investigated the induced effects of JA and SA treatment on *CsLAC* expression levels. Our results showed that under JA treatment, the 9 *CsLAC*s in subclade II (*CsLAC2-1, CsLAC2-2, CsLAC2-5*), subclade IV (*CsLAC4-5*, *CsLAC4-6*, *CsLAC4-8*) and subclade V (*CsLAC5-2*, *CsLAC5-3, CsLAC5-4*) were significantly upregulated, particularly *CsLAC4-5* and *CsLAC4-6*, which were 9.4 and 42.6 times higher, respectively, than the controls (Figure 7A). When tea plants were treated with SA, seven *CsLAC*s (*CsLAC2-2*, *CsLAC3-1*, *CsLAC3-4*, *CsLAC4-6, CsLAC5-2, CsLAC5-3, CsLAC5-4*) and three *CsLAC*s (*CsLAC1-5*, *CsLAC4-1, CsLAC6-1*) were significantly downregulated and upregulated, respectively (Figure 7B). In general, the expression analysis showed that nine *CsLAC*s were up-regulated by JA, but seven *CsLAC*s were down-regulated and three *CsLAC*s were up-regulated by SA. Interestingly, among JA-/SA-responsive genes, the *CsLAC*s (*CsLAC2-2*, *CsLAC4-6*) and subclade V (*CsLAC5-2*, *CsLAC5-3*, *CsLAC5-4*) were concomitantly regulated by JA and SA signaling, but exhibiting opposite expression pattern.

### 2.5. Expression Profiles of CsLACs in Response to Infestation by Herbivores

**Figure 8:**
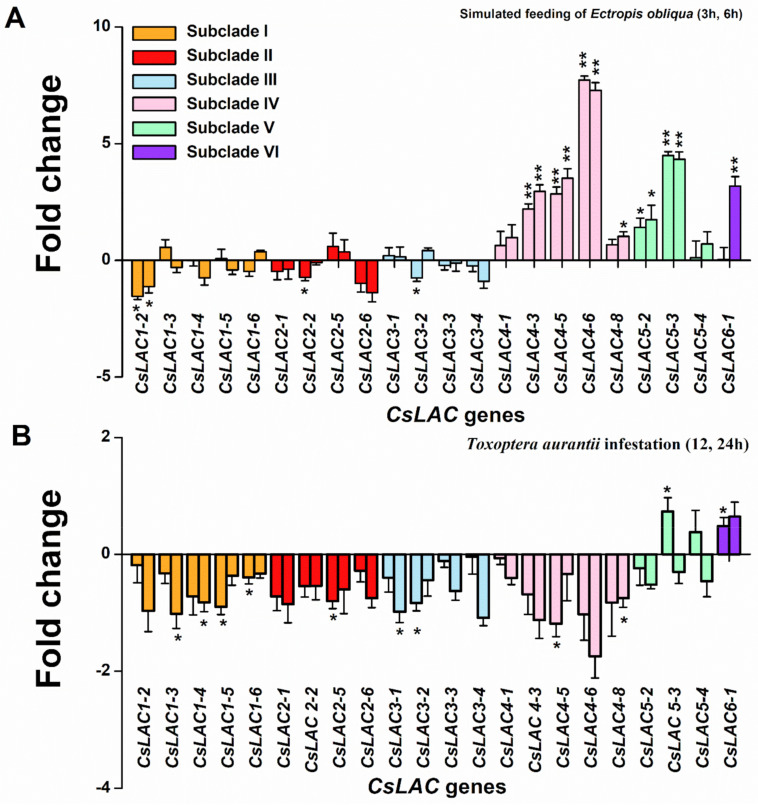
Expression patterns of differentially expressed *CsLAC*s from *C. sinensis* cv ‘Longjing43′ cultivars upon herbivory infestation. The fold change in expression levels (treated group vs. control group) is shown on a log2 scale. (**A**) The change in expression levels of *CsLAC*s in response to simulated *Ectropis obliqua* feeding by RT-qPCR. The left and right bar graphs for each gene represent the fold change after treatment at 3h and 6h, respectively. (**B**) The change in expression levels of *CsLAC*s in response to *Toxoptera aurantii* infestation by RT-qPCR. The left and right bar graphs for each gene represent the fold change after treatment at 12h and 24h, respectively. The asterisks above lines indicate significant differences between the herbivore treatment and the control (*, *p* < 0.05, **, *p* < 0.01, Student’s *t*-test).

Changes in gene expression upon stress are considered as a basic component of plant defense [25]. Many studies have reported that expression levels have been used to analyse the differences in transcriptional profiles of candidate genes under various stress conditions. We also analyzed the expression patterns of *CsLAC*s in leaves following attack by a chewing insect herbivore *Ectropis obliqua* and a pierce-sucking insect herbivore *Toxoptera aurantii,* respectively. Upon simulated feeding of *E. obliqua* at 3 h and 6 h after the start of treatment, the expression levels of 10 genes were changed, while the other 12 were not affected (Figure 8A). The genes in subclade IV (*CsLAC4-3*, *CsLAC4-5*, *CsLAC4-6*, *CsLAC4-8*), subclade V (*CsLAC5-2*, *CsLAC5-3*) and subclade VI (*CsLAC6-1*) were significantly more highly expressed, while the expressions of genes (*CsLAC1-2*, *CsLAC2-2*, *CsLAC3-2*) were downregulated compared to the untreated control. Upon the infestation of *T. aurantii*, the expression levels of genes in subclade I (*CsLAC1-3*, *CsLAC1-4*, *CsLAC1-5*, *CsLAC1-6*) and subclades II, III, IV (*CsLAC2-5*, *CsLAC3-1, CsLAC3-2*, *CsLAC4-5*, *CsLAC4-8*) were significantly downregulated, but expressions of *CsLAC5-3* and *CsLAC6-1* were upregulated (Figure 8B). From the above results, we identified the following two points. First, the results showed that seven *CsLAC*s were up-regulated and three *CsLAC*s were down-regulated under simulated *E. obliqua* feeding, but two *CsLAC*s were up-regulated and nine *CsLAC*s were down-regulated under the infestation of *T. aurantii*, suggesting that the infestation of chewing and pierce-sucking insect herbivores triggered different LAC-responsive defense pathways. Next, among herbivore-responsive genes, the *CsLAC*s (*CsLAC4-3*, *CsLAC4-6*, *CsLAC5-2*, *CsLAC5-3*, *CsLAC6-1*) were upregulated in tea leaves attacked by chewing or/and pierce-sucking insect pests, suggesting their potential broad-spectrum roles in tea resistance against herbivorous pests. However, the expression levels of *CsLAC*s in subclade I (*CsLAC1-3*, *CsLAC1-4*, *CsLAC1-5*, *CsLAC1-6*) and subclades II, III (*CsLAC2-2*, *CsLAC3-1*, *CsLAC3-2*) were downregulated, further suggesting their potential negative roles in tea resistance.

## 3. Discussion

In recent decades, increasing amounts of evidence have demonstrated that plant LAC plays multifaceted roles in development and response to biotic and abiotic stresses. With the completion of whole-genome sequencing in numerous plant species, members of LAC gene families have been identified [8,20]. However, little information is known about the LAC gene family in the tea plant, which is one of the most important woody cash crops in the world. In this study, 43 candidates of *CsLAC*s were identified via a genome-wide search. Large-scale spatiotemporal expression patterns and the potential *CsLAC*-mediated defense response were investigated.

LAC, as a class of polyphenol oxidases, is widely distributed across plant species and encoded by multigene families distributed on different chromosomes. To date, LACs have been characterized at the genome-wide level in many plant species, and their numbers differ greatly, e.g., *A. thaliana* (17 *AtLAC*s on four chromosomes), *Gossypium arboreum* (44 *GaLAC*s on 10 chromosomes), *Citrus sinensis* (24 *CsLAC*s on six chromosomes and chromosome Un), *O. sativa* (30 *OsLAC*s on eight chromosomes), *Glycine max* (93 *GmLAC*s on 19 chromosomes), and *Sorghum bicolor* (27 *SbLAC*s on eight chromosomes) [11,26,27,28,29,30]. The 43 *CsLAC*s in tea plant were unevenly distributed across 10 chromosomes and tended to form five clusters on chromosomes 4, 7, 9, and 10 (Figure 2). Compared with the LAC family in other plant species, a moderate expansion of *CsLAC*s was observed, which was likely consistent with a recent study showing that 28.6% of tea genes occurred in tandem duplication, and most of these genes expanded after a recent tetraploidization event in tea plants [21]. Meanwhile, our results are relatively similar to LAC gene-related studies conducted in *Glycine max*, *Setaria viridis* and *Zea mays* [20,31]. These findings indicated that tandem duplication and chromosomal segmental duplication played the determinant roles in LAC expansion, which seems to follow the most commonly evaluated mechanism underlying gene family expansion [32]. Additionally, the exon-intron structure of 43 *CsLAC*s is similar to that of LAC genes in other plant species [20,27], which indicates that the organization of the gene structure of *CsLAC*s was strongly conserved during evolution. But there were significant variations in the pI values of 43 CsLAC proteins, indicating their functional diversity in the developmental processes and stress responses in tea plants.

Phylogenetic analysis suggested that *CsLAC* members of subclades I, II and III closely clustered with *AtLAC17* and *AtLAC2*, *AtLAC4* and *AtLAC11,* and *AtLAC12* in *Arabidopsis*, respectively. Experimental evidence has demonstrated that these *AtLAC*s were necessary and non-redundant factors for lignification during vascular development [10]. The organ- and development-specific expression patterns of *CsLAC*s were investigated in this study. Generally, most of the *CsLAC*s exhibited leaf- and stem-preferential expression. We therefore speculated that most of the *CsLAC*s of subclades I, II and III were likely involved in constitutive lignin biosynthesis during tea plant growth and development.

During tea leaf development, the lignin content continuously increased, and the genes involved in lignin biosynthesis showed higher expression levels in the leaves with active lignification [29]. Among the selected 23 *CsLAC*s, the regular expression patterns of *CsLAC*s in subclades I, II and IV were observed, whereas *CsLAC*s in subclade III were irregularly expressed on pace with maturity. The expression level of *CsLAC*s in subclades II (from 2nd to 4th leaves) and IV (except for *CsLAC4-1*) decreased on pace with leaf maturity, and this kind of expression pattern was similar to the previously reported 13 *SbLAC*s in *Sorghum bicolor* and one *SofLAC* in sugarcane, which were highly expressed in young internodes but lowly expressed with increasing maturity [26,33]. These observations further reconfirmed the hypothesis proposed previously that this kind of LAC genes may function to polymerize monolignols into oligolignols in early stages of lignification [34]. However, unlike these results, we also found that the expression levels of *CsLAC*s in subclade I (except for *CsLAC1-5*) increased with leaf maturity and decreased when leaves were fully physiologically mature (in 4th leaves). Thus, *CsLAC*s of subclades II, IV and I exhibited opposite expression patterns, implicating potential functional divergence among these subclades during leaf development. Therefore, our results did not fully support the previous hypothesis that LAC might function during early lignification stages, whereas cell wall peroxidases played a role in the follow-up proceedings of xylem development [34]. Thus, we speculated that most *CsLAC*s of subclades II and IV might function during the early lignification process, whereas *CsLAC*s of subclade I were likely involved in the late lignification of xylem development. Thus, the hypothesis we proposed here is that *CsLAC*s in subclades II, IV and I mediated a trade-off for early- and late-stage preferential expression to balance the available resources and facilitate optimal plant development. It is noteworthy that our results were consistent with a recent study on refined model of secondary cell walls lignification, which indicated that LACs were involved in *Arabidopsis* stem lignification throughout growth and development processes [35].

In this study, *cis*-element analysis indicated that many plant hormone, stress and growth responsive elements were identified, which imply that most *CsLAC*s might response to diverse plant hormones and environmental stresses, as well as be involved in diverse processes in plant growth and development. For instance, most *CsLAC*s contained hormone-related elements, including JA and SA signaling elements (Figure 4). JA and SA pathways are thought to play key roles in plant-induced defense responses to biotic and abiotic stresses [36,37,38,39]. Likewise, JA signaling was well-established as the core pathway that regulated tea plant defense against herbivores [24]. Our results showed that JA treatment enhanced the expression of nine *CsLAC*s from subclades II, IV and V, whereas SA treatment decreased the expression of seven *CsLAC*s and enhanced the expression of three *CsLAC*s, suggesting that the JA signaling pathway activated the expression of *CsLAC*s, but the SA signaling pathway was relatively inhibited (Figure 7). This result demonstrates that *CsLAC*s may well be involved in herbivore resistance of tea plants, and *CsLAC*-based defense is positively regulated by the JA signaling pathway. The findings are consistent with the results from our previous studies, which revealed that JA served as a key defense phytohormone that mediated PPO-based resistance via positive regulation of PPO activity along with transcription of *CsPPO*s [40,41]. LAC, known as a kind of PPO, is capable of oxidizing a wide spectrum of aromatic compounds by a radical-catalysed reaction mechanism [6]. Although LAC gene structures and functions are quite different from other kinds of PPOs, all of which might play a similar defensive role in plants via several mechanisms: (1) direct toxicity of phenolic oxidation products, (2) alkylation and reduced bioavailability of cellular proteins to decrease nutritional quality, (3) cross-linking of quinones with other phenolics and lignification of cell walls to form physical barriers, and (4) oxidative stress in the gut lumen of insects [40,42]. Moreover, the expression levels of five *CsLAC*s were upregulated under JA treatment and downregulated under SA treatment in accordance with the antagonism between the two signaling pathways [43], further verifying their primary roles in defense against herbivore in tea plants.

It would be a formidable task to determine the exact role of each *CsLAC* member based on phylogenetic analysis and expression patterns, but, from which many hints can be acquired. LAC is a key enzyme for lignin biosynthesis in the formation of secondary plant cell walls, as it catalyses the final step of monolignol polymerization [26]. In this study, we found that most genes of subclades I, II and III were predominantly expressed in leaves and stems and significantly downregulated by infestation with two important insect pests, *E. obliqua* and *T. aurantii* (Figure 5, Figure 6 and Figure 8). The subclades I and II are closely grouped with *AtLAC17*, *AtLAC2* and *AtLAC4*, *AtLAC11*, the subclade III clustered together with *AtLAC12*. These *AtLAC*s have been verified to be necessary for lignification during vascular development [9,10,13]. Hence, we speculate that most members of subclades I, II and III are mainly involved in constitutive lignin biosynthesis during tea plant growth and development.

Correspondingly, the *CsLAC*s of subclades IV, V and VI may be involved in the response to herbivory attack, which has been supported by several lines of evidence. First, the members of subclades IV (except for *CsLAC4-6*, *CsLAC4-8*), V and VI expressed constitutively at a very low level in leaves (Figure 5), but the expression of *CsLAC*s (*CsLAC4-3*, *CsLAC4-6*, *CsLAC5-2*, *CsLAC5-3*, *CsLAC6-1*) were significantly upregulated in tea leaves attacked by chewing or/and pierce-sucking insect pests (Figure 8). Furthermore, the expression of the subclade IV (*CsLAC4-5*, *CsLAC4-6*, *CsLAC4-8*) and subclade V (*CsLAC5-2*, *CsLAC5-3*, *CsLAC5-4*) were significantly upregulated by exogenous application of JA (Figure 7). These results clearly indicated that some *CsLAC*s in subclades IV, V and VI exhibited stress-inducible expression patterns under biotic and abiotic stresses. Second, *CsLAC4-6* and *CsLAC4-9* were clustered with *Arabidopsis AtLAC14* and *AtLAC15*, which have been previously reported to take part in the polymerization of phenolic compounds. Furthermore, *AtLAC14* and *AtLAC15* were phylogenetically related to *GhLAC1* and *GhLAC15* in upland cotton, which have been demonstrated to be involved in positively regulating defense-induced lignification in the cell wall to enhance the broad-spectrum biotic stress response [12,13]. A recent study demonstrated that the overexpression of poplar *PeuLAC2*, which phylogenetically clustered with *AtLAC14* and *AtLAC15* of *Arabidopsis*, altered the xylem structure of plants, including thickening the secondary cell wall (SCW) and increasing the fiber cell length and stem tensile strength, and thereby mediated stronger antioxidant response and greater drought tolerance [44]. In our study, most *CsLAC*s in subclade IV were remarkably upregulated in *E. obliqua* simulated feeding leaves. Third, *CsLAC*s of subclade V clustered together with wound-induced *AtLAC8* and *AtLAC9* (Figure 1), implying that they may have similar functions in response to mechanical damage [8]. There have been few reports of the defense functions of LACs in subclade V under herbivory infestation in other plant species thus far. Our finding has obviously shown that *CsLAC*s of subclade V are potentially crucial for the herbivore resistance of tea plants, but the in-depth mechanism by which members contribute to tea plant resistance needs to be fully clarified. Based on these results, the study showed that the some *CsLAC*s of subclades IV, V and VI were stress-induced and likely involved in induced lignin biosynthesis to facilitate tea plant resistance against herbivory.

In this study, herbivore attack enhanced the expression of most *CsLAC*s in subclades IV, V and VI, but suppressed most genes in subclades I, II and III, which exhibited an opposite expression pattern. These results seems to obey the law that plants suffer limited resource reallocation to facilitate the prioritization of defense towards growth to survive during herbivore attack [4,45], and may account for the incompatibility between growth and defense programmes, known as the “growth-defense trade-off” phenomenon established upon plant-herbivore interactions [5,46,47,48,49,50]. Altogether, our findings provide one *CsLAC*s-based explanation for the growth-defense trade-off in tea plant at the molecular level.

**Figure 9:**
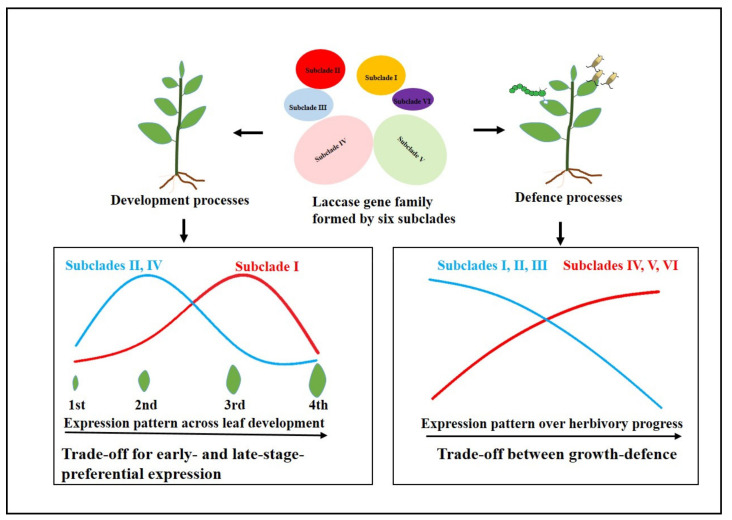
Schematic diagram showing LAC gene family-mediated trade-offs during tea plant development and defense processes. The curve (red) indicates transcripts that increase with leaf development or damages under herbivore attack. The curve (blue) represents transcripts that decrease with the progress of leaf development or herbivory.

In summary, 43 *CsLAC*s were investigated through genome-wide analysis of *C. sinensis*. Considering these findings, we shed new light on this phenomenon and propose a simplified model in Figure 9. A spatiotemporal expression analysis indicated that most *CsLAC*s displayed tissue-special or stage-preferential expression patterns, which suggesting a trade-off for early- and late-stage-preferential expression patterns during leaf development. According to expression profiles of *CsLAC*s responding to biotic and abiotic stress, a delicate trade-off between growth and induced defense was presented in a manner that prioritizes defense over growth. Additionally, our results provide evidence for the defense function of *CsLAC*s in tea plants, which was positively regulated by the JA signaling pathway. More in-depth investigation to delineate the upstream regulation and downstream targets mediating herbivore defense should be carried out in the future.

## 4. Materials and Methods

### 4.1. Plants and Insects

The plant materials were collected from 3-year old tea plants (*C. sinensis* cv. Longjing 43), growing in a greenhouse programmed at 25 ± 2 °C, 14 h light (L): 10 h dark (D), and 60–80% relative humidity (RH). Healthy plants at same growth stage were chosen for experiments.

The tea looper (*Ectropis obliqua* Warren) and tea aphid (*Toxoptera aurantii* Boyer) were collected from the tea plantation of the Tea Research Institute of the Chinese Academy of Agricultural Sciences (TRI, CAAS, N 30° 10′, E 120°5′), Hangzhou, China and fed with fresh tea shoots in the controlled climate room at 26 ± 2 °C, 70 ± 5% RH, and a photoperiod of 14:10 h (L: D). Newly hatched larvae/nymphs were fed in 75 × 75 × 75 cm net cages with fresh tea shoots. Over one generation, three-instar larvae of *E. obliqua* and mixed age nymphs of *T. aurantii* were used for plant treatments.

### 4.2. Regurgitant Collection

Following the method proposed by Yang et al. [51], regurgitant was collected from the oral cavity of *E. obliqua* with a P200 Pipetteman (Gilson, Middleton, WI, USA) and stored at −80 °C.

### 4.3. Confirmation of the CsLACs Gene Family

Total RNA extractions were performed with the TRIzol™ kit according to the manufacturer’s instructions (TIANGEN, Beijing, China). RNAs were reverse-transcribed using PrimeScript RT Master Mix (TaKaRa Bio, Dalian, China). The cDNA fragments were obtained by transcriptome. The 5′ and 3′ sequences of *CsLAC*s were acquired by rapid amplification of cDNA ends (RACE) using the manufacturer’s protocol (SMARTer^®^ RACE 50/30Kit, Clontech Lab, Inc., Mountain View, CA, USA). The primers for gene cloning of 30 *CsLAC*s used are listed in Supplementary Table S2. For another 13 candidate *CsLAC*s, BLASTp was performed in the *C. sinensis* genome using the AtLAC protein sequences as queries and sequences with E-value < 10^−20^ were selected. All the selected genes were checked against information from the NCBI database. The sequences with essential conserved domains of multicopper oxidase type were deemed as candidate *CsLAC*s.

### 4.4. Phylogenetic Analysis and Distribution Pattern of CsLACs

A neighbor-joining phylogenetic tree was constructed by MEGA (http://www.megasoftware.net/ accessed on 18 February 2020) using 60 protein sequences from *A. thaliana* (17) and *C. sinensis* (43), with bootstrap tests for 1000 replicates. The sequences form *A. thaliana* used for phylogenetic analysis were listed in Supplementary Table S1. The chromosomal location map of *CsLAC*s was generated using MapInspect software (http://mapinspect.software.informer.com/ accessed on 18 February 2020).

### 4.5. Analysis of Gene Structure, Conserved Motif and Cis-Elements

The exon-intron structures of *CsLAC*s were determined using Gene Structure Display Server (GSDS v2.0; http://gsds.cbi.pku.edu.cn/ accessed on 28 May 2020). The molecular weight (Mw) and theoretical isoelectric (pI) points of CsLACs were calculated by ExPASy (http://web.expasy.org/compute_pi/ accessed on 28 May 2020). Subcellular localization pattern of CsLACs were predicted using web based tool TargetP 1.1 server. The promoter sequences of *CsLAC*s were investigated for with PlantCARE (http://bioinformatics.psb.ugent.be/webtools/plantcare/html/ accessed on 28 May 2020).

### 4.6. Plant Treatments

#### 4.6.1. Different Tissues and Leaves at Different Stages of Maturity

Various tissues, including leaves, stems, roots, flowers and seeds, were harvested. The 1st, 2nd, 3rd and 4th leaves were collected from the same branch of tea plant. Six replications were collected and all samples were quickly frozen in liquid nitrogen and then stored at −80 °C for further study.

#### 4.6.2. Simulated Feeding of *E. obliqua*

The mechanical damage was made by a fabric pattern wheel following the method described in the reference of Li et al. [52]. The second fully expanded leaves were used in the following treatments. Each leaf was rolled six times, and 15 μL regurgitant of *E. obliqua* was painted to the puncture wounds. The second leaves kept intact were used as controls. All the second leaves were harvested at 3 h and 6 h after the start of treatment. Eight replications were carried out.

#### 4.6.3. *T. Aurantii* Infestation

In this study, the *T. aurantii* in 3rd, 4th, and 5th instar nymphs were used for plant treatments. Fifty aphids were inoculated on the tender buds and 1st leaves, while the 2nd leaves were covered with fine-mesh sleeves to keep them from infestation and contamination of honeydews. Second leaves covered with fine-mesh sleeves from plants without aphids were used as controls. The 2nd leaves were harvested at 12h and 24h after the start of treatment. Six replications were carried out.

#### 4.6.4. JA and SA Treatment

JA and SA (Sigma Aldrich, Saint Louis, MO, USA) were dissolved in ethanol to make 100 mM stock solutions. The final spray concentrations were 100 µM for JA, and 1 mM for SA, which were diluted in distilled water containing 0.02% (*v*/*v*) Tween 20. For the control, 0.02% (*v*/*v*) Tween 20 in distilled water containing 0.01% ethanol was applied. The solution was generously sprayed onto the surfaces of the tea leaves until liquid dripped off the leaves. After spraying, the plants were covered with sealed square plastic containers (65 cm × 65 cm × 65 cm). The second leaves were harvested at 6 h and 24 h after the start of treatment. Eight replications were carried out.

### 4.7. RNA Sequencing and Data Processing

RNA samples of tea leaves were prepared for RNAseq analysis. Paired end of 2 × 150 bp sequencings were carried on Illumina HiSeq platform commercially provided by Metware Biotechnology Co., Ltd. (Wuhan, China). Fastp was used to further improve the quality of sequence data and assess the quality of clean reads [53]. Then the clean reads were mapped to the transcriptome extracted from tea genome data [22,54]. DEGs (differential expressed gene) were identified by DESeq2 with the criteria of absolute value of FDR (false discovery rate) < 0.05 [55].

### 4.8. qRT-PCR Analysis

The qRT-PCR reactions were performed on a LightCycle^®^ 480 Real-Time PCR System (Roche Diagnostics, Mannheim, Germany) with 10μL reaction mixture. A five-fold dilution series of cDNA was used as a template for each treatment using a linear regression model to create the standard curves. The relative expressions were calculated by the standard curve method. All the primers for *CsLAC*s and the reference genes used are listed in Supplementary Tables S3 and S4.

### 4.9. Data Analysis

All data were analyzed with SPSS software version 20 (SAS Institute, Inc., Cary, NC, USA, http://www.sas.com/ accessed on 1 January 2021). Differences in data among different treatments were analyzed using one-way ANOVA. If the ANOVA analysis was significant (*p* < 0.05), Turkey’s honestly significant difference (HSD) post-hoc test was used to detect differences between groups. Student’s *t*-test was used for comparing the difference between two treatments. When necessary, data were log-transformed to meet requirements for the homogeneity of variance.

## Figures and Tables

**Table 1:** Identification and analyses of *CsLAC*s.

Name	Accession Number	Intron	Extron	ORF Size (bp)	Protein			Sub-Cellular Localization
					Amino Acid Residues	MW (Da)	pI	
*CsLAC1-1*	CSS0050170	6	7	1734	578	64.230	9.54	Secretory
*CsLAC1-2*	CSS0030617	5	6	1710	569	62.934	9.47	Secretory
*CsLAC1-3*	CSS0040822	5	6	1740	580	63.814	9.30	Secretory
*CsLAC1-4*	TEA004738.1	5	6	1734	578	63.801	8.81	Secretory
*CsLAC1-5*	CSS0032027	5	6	1737	579	64.134	8.90	Secretory
*CsLAC1-6*	CSS0029337	6	7	1755	585	64.656	9.14	Secretory
*CsLAC1-7*	CSS0045289	7	8	1755	584	64.398	9.02	Secretory
*CsLAC2-1*	CSS0030904	6	7	1674	558	60.821	9.34	Secretory
*CsLAC2-2*	CSS0014129	6	7	1665	554	61.137	8.93	Secretory
*CsLAC2-3*	CSS0015036	5	6	1668	555	61.106	8.34	Secretory
*CsLAC2-4*	CSS0047304	6	7	1713	571	63.545	8.99	Secretory
*CsLAC2-5*	CSS0001101	5	6	1692	564	63.073	8.25	Secretory
*CsLAC2-6*	CSS0005481	5	6	1671	557	62.160	9.12	Secretory
*CsLAC3-1*	CSS0035921	5	6	1728	575	62.560	6.13	Secretory
*CsLAC3-2*	CSS0017559	4	5	1746	581	63.949	9.18	Secretory
*CsLAC3-3*	CSS0041657	5	6	1722	574	63.758	6.30	Secretory
*CsLAC3-4*	CSS0009670	5	6	1719	573	64.121	9.05	Secretory
*CsLAC4-1*	NOT FOUND	-	-	1335	444	49.38	5.51	Non-secretory
*CsLAC4-2*	CSS0038356	4	5	1821	606	67.629	5.26	Secretory
*CsLAC4-3*	CSS0043663	6	7	1773	591	66.744	5.27	Secretory
*CsLAC4-4*	CSS0043918	5	6	1707	569	63.872	8.95	Secretory
*CsLAC4-5*	CSS0007135	5	6	1683	561	62.828	5.16	Secretory
*CsLAC4-6*	CSS0022921	5	6	1767	589	66.331	7.62	Non-secretory
*CsLAC4-7*	CSS0004662	5	6	1713	571	63.469	7.22	Secretory
*CsLAC4-8*	CSS0047533	3	4	1701	567	62.980	8.52	Secretory
*CsLAC4-9*	CSS0036236	5	6	1638	545	61.148	6.09	Non-secretory
*CsLAC4-10*	CSS0025249	6	7	1544	522	59.050	5.33	Secretory
*CsLAC4-11*	CSS0039645	5	6	1701	566	63.180	8.30	Secretory
*CsLAC4-12*	CSS0046037	5	6	1425	474	53.109	5.05	Non-secretory
*CsLAC5-1*	CSS0013370	5	6	1824	608	67.871	6.53	Non-secretory
*CsLAC5-2*	CSS0010920	5	6	1695	565	62.439	6.33	Secretory
*CsLAC5-3*	CSS0010479	5	6	1698	565	62.148	6.18	Secretory
*CsLAC5-4*	CSS0020412	5	6	1707	569	62.319	7.34	Secretory
*CsLAC5-5*	CSS0045107	5	6	1698	565	62.463	6.33	Secretory
*CsLAC5-6*	CSS0048878	6	7	1602	533	58.490	6.14	Secretory
*CsLAC5-7*	CSS0008882	5	6	1695	564	62.714	6.08	Secretory
*CsLAC5-8*	CSS0010391	5	6	1701	566	62.778	6.59	Secretory
*CsLAC5-9*	CSS0030703	5	6	1698	565	62.411	6.88	Secretory
*CsLAC5-10*	CSS0044116	5	6	1698	565	62.360	8.31	Secretory
*CsLAC5-11*	CSS0013475	5	6	1719	572	63.498	7.07	Secretory
*CsLAC5-12*	CSS0023848	5	6	1710	569	62.247	6.71	Secretory
*CsLAC6-1*	TEA021330	4	5	1737	579	64.576	6.82	Secretory
*CsLAC6-2*	CSS0019151	5	6	1725	574	63.731	6.36	Secretory

## Data Availability

The data that support the findings of this study are available from the corresponding author upon reasonable request.

## Supplementary Materials

The following are available online at https://www.mdpi.com/article/10.3390/ijms222212554/s1, Figure S1: Multiple sequence alignment of subgroup 1-6 of CsLAC gene family, Table S1: The gene ID used for phylogenetic analysis, Table S2: Primers used for cloning laccase genes in *Camellia sinensis*, Table S3: Primers used for qRT-PCR, Table S4: Sequence information of the reference genes used under different treatments.
