# Supplementary material for: The Laccase Gene Family Mediate Multi-Perspective Trade-Offs during Tea Plant (Camellia sinensis) Development and Defense Processes
Source: Int J Mol Sci. 2021 Nov 21;22(22):12554. doi: 10.3390/ijms222212554 (PMC8618718; doi:10.3390/ijms222212554)
Supplement: Supplementary file 1 [file ijms-22-12554-s001.zip › 1414730-supplementary.pdf]

International Journal of *Molecular Sciences*

MDPI

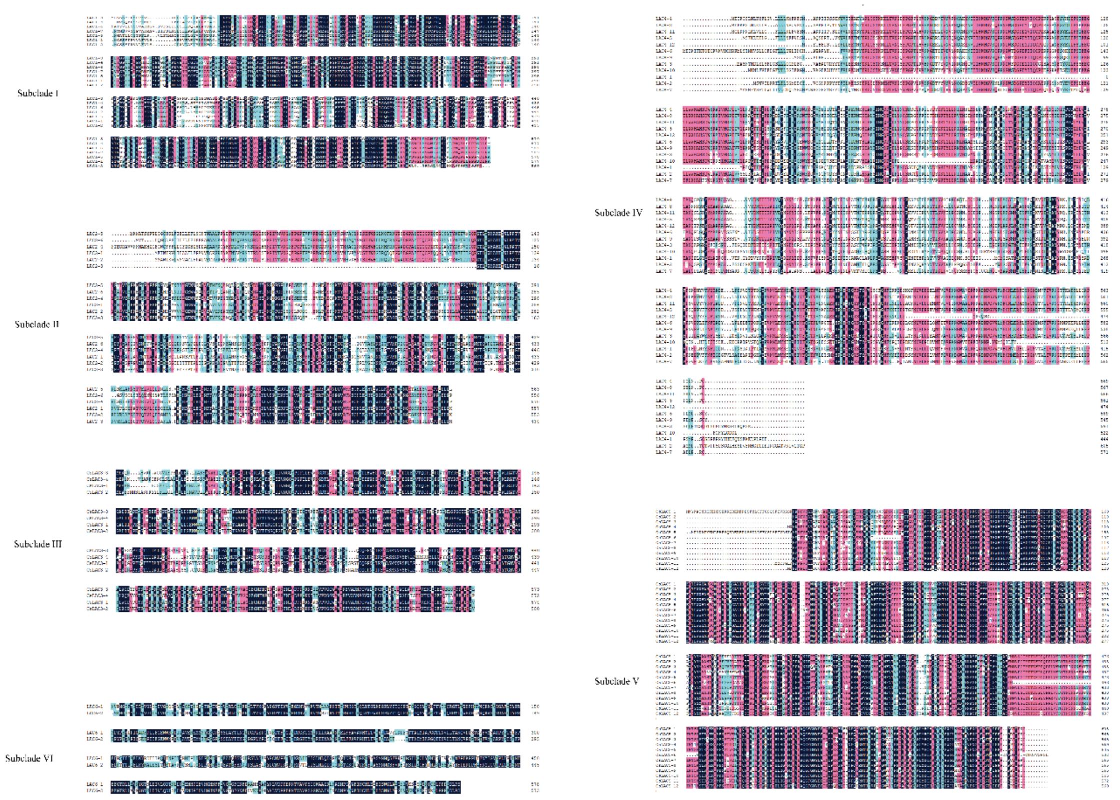

Figure S1. Multiple sequence alignment of subgroup 1-6 of *CsLAC* gene family. Sequence alignment among *CsLAC*s showed the highest identity of 80.99% in group I, identity of 80.44%, 80.25%, 71.41% and 71.16% respectively in group III, group V, group II and group VI. There was the lowest similarity in laccase protein sequences from group IV, sharing an identity of 59.25%.

Table S1. The gene ID used for phylogenetic analysis.

Gene	Resource	Locus number
*At*LAC1	*Arabidopsis thaliana*	At1g18140
*At*LAC2	*Arabidopsis thaliana*	At2g29130
*At*LAC3	*Arabidopsis thaliana*	At2g30210
*At*LAC4	*Arabidopsis thaliana*	At2g38080
*At*LAC5	*Arabidopsis thaliana*	At2g40370
*At*LAC6	*Arabidopsis thaliana*	At2g46570
*At*LAC7	*Arabidopsis thaliana*	At3g09220
*At*LAC8	*Arabidopsis thaliana*	At5g01040
*At*LAC9	*Arabidopsis thaliana*	At5g01050
*At*LAC10	*Arabidopsis thaliana*	At5g01190
*At*LAC11	*Arabidopsis thaliana*	At5g03260
*At*LAC12	*Arabidopsis thaliana*	At5g05390
*At*LAC13	*Arabidopsis thaliana*	At5g07130
*At*LAC14	*Arabidopsis thaliana*	At5g09360
*At*LAC15	*Arabidopsis thaliana*	At5g48100
*At*LAC16	*Arabidopsis thaliana*	At5g58910
*At*LAC17	*Arabidopsis thaliana*	At5g60020

Table S2. Primers used for cloning laccase genes in *Camellia sinensis*.

Primers	Purpose	Primer Sequence (5′→3′)
1-1RACE3-1	5′-RACE	GCGGCAAGTGTTGGTATTAGCAGGGG
1-1RACE3-2	5′-RACE	AGGGATGACGAAATGGGTAAGGGACA
1-2RACE5-1	5′-RACE	GTGGTATTCGAGGATTGCGGCGGTAG
1-2RACE5-2	5′-RACE	CTCGGCGGATTGCCCGTGTAGTTGA
1-2RACE3-1	3′-RACE	GTGGTTGAAGCGGACGCAATCTAT
1-2RACE3-1	3′-RACE	ACCACTGATTTTCCAGCGAACCCG
1-3RACE5-1	5′-RACE	TTCTCCGCCCCCAAAATACTGGTATCCT
1-3RACE5-2	5′-RACE	TGGACAGTTTTAGGGACCTTATTGGCAG
1-4RACE3-1	3′-RACE	ATTGAAAAGCCTCGCATCCCCTAAA
1-4RACE3-2	3′-RACE	CGCCGAACAACACGATGGTGAGTAA
1-5RACE5-1	5′-RACE	CACTCTCCAAAGATGATGGGAACCT
1-5RACE5-2	5′-RACE	GCCAGTTTGTATGGGGCATTGAGTT
1-5RACE3-1	3′-RACE	TGGTCCTATAATCATCCGTCCCAAACA
1-5RACE3-2	3′-RACE	GGAGGTTCCCATCATCTTTGGAGAGTG
1-6RACE3-1	3′-RACE	GGTGGCATTCATGGGAGTTATCGT
1-6RACE3-2	3′-RACE	TTCCTCAACCAGCACTTGCCACGG
1-7RACE5-1	5′-RACE	TGGGGGACATTGGCAGGGTATTGAGC
1-7RACE5-2	5′-RACE	TGGCAAGGCTGAGTGAAGAAGTGGTG
1-7RACE3-1	3′-RACE	GACCGACACGCTTCTCATTACTCCCG
1-7RACE3-2	3′-RACE	GTGCCACATTCTTGATGATTGCCAGG
2-1RACE5-1	5′-RACE	TTGCCATTGTCCACCAAGAATGCCATC
2-1RACE5-2	5′-RACE	CCATCTTTAGTCCCCATGATGTGTGCA
2-2RACE3-1	3′-RACE	ACCCCGCCTCCTAAAAACGCCACCCCAG
2-2RACE3-2	3′-RACE	GAGTTCCGTCTGGAGGATGGACTGCTAT
2-3RACE5-1	5′-RACE	CCACCGCCACCACACTGTCCATAAA
2-3RACE5-2	5′-RACE	CTGGCGGTTAGGAGGGCATTTGTGG
2-3RACE3-1	3′-RACE	AGACCACAAATGCCCTCCTAACCGC
2-3RACE3-2	3′-RACE	CTTTATGGACAGTGTGGTGGCGGTG
2-4RACE5-1	5′-RACE	AAGTCACTGGAGGGCGGTCTGGG
2-4RACE5-2	5′-RACE	TGGTGTGCCCAGCGATGGCGAAG
2-5RACE5-1	5′-RACE	GTGGTCCTGGTTTCCCATTGATTGT
2-5RACE5-2	5′-RACE	TTTGGAATGGCACAGCCTGCTCACA
2-5RACE3-1	3′-RACE	TTCCTTTCTTGCCACACTAACGCTGC
2-5RACE3-2	3′-RACE	TGTTCCTACTGGCGGTTGGACTGCTA
2-6RACE5-1	5′-RACE	CCCTGGGAACATCCCATTCACTGTCA
2-6RACE5-2	5′-RACE	TGCTCACATTCTTCACTTGTATCTGA
2-6RACE3-1	3′-RACE	ACAAGTGAAGAATGTGAGCAGATTGTG
2-6RACE3-2	3′-RACE	TGACAGTGAATGGGATGTTCCCAGGGC
3-1RACE5-1	5′-RACE	GGATTGAAGTTTCCAAAGCCCTGTGCGA
3-1RACE5-2	5′-RACE	CTGCCCCAGTTCTTGTAGCCTGCCTCAC
3-2-F	Clone	CTTGGGTTGTAGTAGTTTC
3-2-R	Clone	AACCAAAACCAAAACCACCC
3-3RACE3-1	3′-RACE	CCCCAGCCTTGCTCTATTTGCTCTCAT
3-3RACE3-2	3′-RACE	AGACAGAGGGGAACGCCGTGGGCAGAC
3-4RACE5-1	5′-RACE	CCCTGGATTATCAGCGACAAACCG
3-4RACE5-2	5′-RACE	TGCCCATCCACCAACAGGGACATC
4-1RACE5-1	5′-RACE	GGAACACGAAAGGTATTCACTTGCGG
4-1RACE5-2	5′-RACE	GCAGGACATTGCTTTCTTGGAACACT

4-2RACE5-1	5′-RACE	TTGTCTCTGGTGTCCCGCCATCTCTT
4-2RACE5-2	5′-RACE	TTCCTGCGTCCAACACATTAGTCCCT
4-2RACE3-1	3′-RACE	GCCTGAATACATCACACAATGCGGAA
4-2RACE3-2	3′-RACE	CAAGGGACTAATGTGTTGGACGCAGG
4-3RACE5-1	5′-RACE	GACCCCAACAGTGTTCAACTCAGGTG
4-3RACE5-2	5′-RACE	GACCCAACGACATAAAAACTGAACCC
4-3RACE3-1	3′-RACE	GGGTTCAGTTTTTATGTCGTTGGGTC
4-3RACE3-2	3′-RACE	TCCACCTGAGTTGAACACTGTTGGGG
4-4RACE5-1	5′-RACE	TGAAAAAATAAGGTGGTTGACTCGGAA
4-4RACE5-2	5′-RACE	TCACCATTGGGTCCATCACAAGAACTG
4-5RACE5-1	5′-RACE	TGATGATTTGCGATGGAGAAAAAGAGAA
4-5RACE5-2	5′-RACE	GCCACCATAGGGTTCCTTCCTCTGTTGA
4-6RACE5-1	5′-RACE	GGATTGGGCACTGGGTAATGTAGGAA
4-6RACE5-2	5′-RACE	GGTGGCATTGTAGGGAGATTGGTTGG
4-7RACE3-1	3′-RACE	CTCCGCCAAGCCCATCCCTACCA
4-7RACE3-2	3′-RACE	GGTTCTGACGGAAACTAAATACACACG
4-8RACE5-1	5′-RACE	GACCCAAGCCAGTATTCCTCGTGTGAC
4-8RACE5-2	5′-RACE	CATCATTGTCGTGAAAGCCTGGAAAGA
4-8RACE3-1	3′-RACE	CAACAATCTTTCCAGGCTTTCACGACA
4-8RACE3-2	3′-RACE	ACCGATTTCATCCTCCACAAGGTTCAT
5-1RACE5-1	5′-RACE	TGACATTGTTGACAAAGGCTTCCATCA
5-1RACE5-2	5′-RACE	ATAGAACTTGTGGGCTGTTGGTGTGTC
5-1RACE3-1	3′-RACE	ATGACACACCAACAGCCCACAAGTTCT
5-1RACE3-2	3′-RACE	CCCATGCGAAGCAGGTTCCAACTGTAG
5-2RACE5-1	5′-RACE	CGGGAGCAATAACAATGAAGTCGGTGA
5-2RACE5-2	5′-RACE	TTGGAACTGTTAGGAGCACCACCGC
5-2RACE3-1	3′-RACE	TCTCCAACGACTATGCCGCAATCAA
5-2RACE3-2	3′-RACE	ACCGACTTCATTGTTATTGCTCCCG
5-3-F	Clone	CGATCTCATCGTTGTATGAAATAC
5-3-R	Clone	AGCAATACGGGAAAAATGTTAG
5-4RACE3-1	3′-RACE	TTCGTACCCATTCCCAAAACCGCA
5-4RACE3-2	3′-RACE	TGCCACTCCATCAACCCCACAGA
6-1RACE5-1	5′-RACE	GATTAGGACCCGACCCGCTTTTCAC
6-1RACE5-2	5′-RACE	CCGTTGAGTCAGGCACTTGATTCGC
6-1RACE3-1	3′-RACE	GAATCAAGTGCCTGACTCAACGGG
6-1RACE3-2	3′-RACE	GGGACAAGGCTTTTGGAGGTGAAC

Table S3. Primers used for *q*RT-PCR.

Name	Forward primer (5′→3′)	Reverse primer (5′→3′)
LAC1-2	AGACGTGTCAAGGACCCAAC	ATGGAGATGCAAAGGGTGAG
LAC1-3	ACAACTGCTCTCCTCCAGTC	GTTAGGTGGTGTCCCGGTAT
LAC1-4	CTCAAAACCAAACCGCATTT	GGACGTTTGCTGGGAATTTA
LAC1-5	ACAAGATTGTGCCACACCAA	ATATGGGCATGCCAAAAGAG
LAC1-6	GGACAGACCACAAACGTCCT	ATTGGCAGGGTATTGAGCAC
LAC2-1	GCCACGGTTCAACTTGTTTT	ATGTGTGCACCTCCAAATGA
LAC2-2	CCTCGGTGTCAATCCTTGTT	TTCAGGGGCTATCATTCCAG
LAC2-5	CCACACTAACGCTGCATTGA	CCCGGGAACATTCCATTCAC
LAC2-6	TCCATTGGCATGACTTGAAA	GTGTGCATGCCACCATAAAG
LAC3-1	GCACCTGTCAAGGTCCAAAT	AGTGACCGGCTCACATTACC
LAC3-2	CCCTTCACCACCTCAGTCAT	GGCTTTCATGGGGTATCTGA
LAC3-3	TCCTTCCAATGTCGAAGTCC	AAGGTAGGTTGCCACAATGC

LAC3-4	TCCCGAGAAGGAAAAGGAAT	GACCCTGGAACCAAACTTCA
LAC4-1	CGGCAAAGAACACCCTGTAT	TTCGCATTGACTCTTGTTGC
LAC4-3	TATCACTGCAAGCGACCAAG	AATCTGATGACTGCCCAACC
LAC4-5	TGCATGTTCACAAGGGTGAT	CCATGGATTTCTTGGTTGCT
LAC4-6	ACCCAGGAGTGTGGCTTATG	TGGAGGCAGCAGTTTTTCTT
LAC4-7	GGGACAGTTACCACCGAGAA	CAAGTTCAAATTGGCGGTTT
LAC4-8	TGGCTTTGCTCTTGAATGTG	TGCTCTACTCCATGCCAGTG
LAC5-2	TGACCTTCGTTTCTTTGGCTCT	CAACATCAGCGGAGGTGAGT
LAC5-3	GTTGAAAACCGACCAGCCAC	CCCAATGAGGCGCATTTACG
LAC5-4	TTATGAATGGGCCTCACTGG	TTGCACCTAATCGTTGCTTG
LAC6-1	CTTGCTGCCAGGAACACAAC	CTCCTGTGATTGGGCACTGT

Table S4. Sequence information of the reference genes used under different treatments.

**NO**	**Treatments**		**Reference genes for each treatment**	**GeneBank Accession Number**	**Primer sequence (5′–3′) forward/reverse**
	**Names**	**Organs**			
1	tea leaves at different stages of maturity	1st leaves<br>2nd leaves<br>3rd leaves<br>4th leaves	*CsTBP*	AT1G55520	GGCGGATCAAGTGTTGGAAGGGAG<br>ACGCTTGGGATTGTATTCGGCATTA
2	*T. aurantii* infestation	2nd leaves	*CsACTIN1*	KA280216.1	TGGGCCAGAAAGATGCTTATGTAGG<br>ATGCCAGATCTTTTCCATGTCATCC
3	*E.grisescens* infestation	2nd leaves	*CsGAPDH1*	KA295375.1	TTTTTGGCCTTAGGAACCCAGAGG<br>GGGCAGCAGCCTTATCCTTATCAGT
4	JA treatment	2nd leaves	*CsGAPDH1*	KA295375.1	TTTTTGGCCTTAGGAACCCAGAGG<br>GGGCAGCAGCCTTATCCTTATCAGT
5	SA treatment	2nd leaves	*CsTBP*	AT1G55520	GGCGGATCAAGTGTTGGAAGGGAG<br>ACGCTTGGGATTGTATTCGGCATTA
